# Challenges of Diagnosing Primary Ewing’s Sarcoma in the Small Intestine of the Elderly: A Case Report

**DOI:** 10.3389/fonc.2021.565196

**Published:** 2021-07-09

**Authors:** Jianchuan Yang, Hong Wei, Yucheng Lin, Ning Lin, Songsong Wu, Xunbin Yu

**Affiliations:** ^1^ Department of Ultrasonography, Fujian Provincial Hospital, Shengli Clinical Medical College, Fujian Medical University, Fuzhou, China; ^2^ Department of Cadre Health Care Office, Fujian Provincial Hospital, Shengli Clinical Medical College, Fujian Medical University, Fuzhou, China; ^3^ Department of Ultrasonography, Affiliate Fuzhou First Hospital, Shengli Clinical Medical College, Fujian Medical University, Fuzhou, China; ^4^ Department of Pathology, Fujian Provincial Hospital, Shengli Clinical Medical College, Fujian Medical University, Fuzhou, China

**Keywords:** Ewing’s sarcoma, extraosseous Ewing’s sarcoma, primitive intestine tumor, elderly, ultrasonography

## Abstract

Extraosseous Ewing’s sarcoma (EES) is a malignant tumor that is classified as a rare disease. EES is common in children and adolescents, with a rarer incidence being present in the elderly. ES of the primary intestine is rare, with only a few reports described in the literature. Here we report a case of a 69-year-old male patient who was experiencing abdominal pain for over 3 months. Ultrasonography (US) revealed a solid hypoechoic lesion with multiple irregular necrotic areas in the left lower abdomen close to the dome of the bladder. Contrast-enhanced ultrasonography (CEUS) showed that the lesion exhibited heterogeneous enhancement and quick peripheral enhanced tissue wash-out classifying this mass as malignant. PET–CT showed a high metabolic mass in the lower abdomen, multiple metabolic nodules in the mesentery, considered as a small intestinal stromal tumor with lymph nodes metastasis, and that a diagnosis of lymphoma should be excluded. Esophagogastroduodenoscopy performed at another hospital 1 month prior to CT showed an abnormal density in the pelvic cavity that was considered as a colonic diverticulum with an abscess. The endoscopy showed no obvious occupying lesions. The mass was removed and postoperative pathology confirmed ES of the small intestine. The patient avoided receiving chemotherapy. After 2 months, skull metastasis was diagnosed and surgical intervention was done. His survival was only six months after the second surgery. To our knowledge, our case is the first report of ultrasound and CEUS manifestation of EES in the small intestine in elderly.

## Introduction

The Ewing’s sarcoma (ES) family of tumors is highly aggressive and includes the extraosseous ES (EES), peripheral primitive neuroectodermal tumor (PNET), Askin’s tumor and atypical ES (1). ES mainly occurs in the pelvic region or proximal long bone tissues in 10–20 year old adolescents (2, 3). There are only a few reports of EES in adolescents in the literature; EES in the elderly with primary ES of the small intestine is extremely rare (4–8). There are rare reports on the ultrasound and CEUS imaging features of EES, most of which are CT, MRI, and PET/CT images. It is challenging for physicians to come up with a pre-operative diagnosis since ESS has non-specific imaging features (9, 10). Here, we report a highly aggressive case of primary ES in the small intestine of a 69-year-old man with a short survival.

## Case Presentation

**Figure 1:**
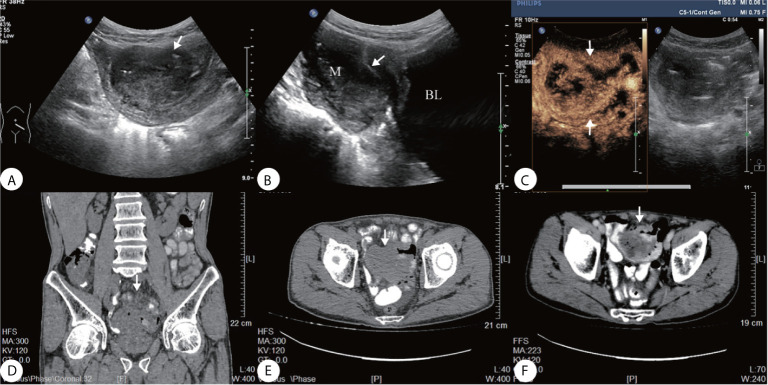
**(A)** Ultrasound showed a well-defined heterogeneous hypoechoic mass on the left lower abdomen, **(B)** the mass close contact with the bladder wall (arrow); **(C)** Contrast-enhanced ultrasonography (CEUS) presented irregular necrotic areas, heterogeneous enhancement in the arterial phase with quick wash-out (54 s); **(D–F)** Abdominal CT showed a hypodense solid lesion in the wall of an ileal loop, with areas of necrosis within (arrow).

A 69-year-old man presented a 3-month history of persistent dull pain in the left lower abdomen. The patient experienced occasional diarrhea, slightly black stools, a poor appetite, fatigue, and 20-lb weight loss. There was no nausea, vomiting, fever, or night sweats. The patient denied any personal or family history of cancer. Before being transferred to our hospital, the patient underwent a gastrointestinal endoscopy that showed no abnormalities. Abdominal CT revealed a large, irregular mass in the pelvic cavity (**Figures 1D–F**) that was considered as a intestine diverticulum with an abscess. He was suspected to have an inflammation and was treated with a two-week course of antibiotics in another medical facility. However, the antibiotics did not relieve his symptoms. His hemoglobin level was 111 g/L (standard 135–170 g/L), and occult blood (OB) test was positive. Biochemical infection screening and tumor markers (CEA, AFP, CA199, CA724, CA125) were all normal on admission. On physical examination, he was found to have a well-defined soft mass on the left lower abdomen, poor mobility, slight tenderness. Ultrasonography revealed a 6.1 3.8 × 4.2 cm irregular, heterogeneous hypoechoic mass in the left lower abdomen (**Figures 1A, B** **)**. The tumor contained multiple necrotic areas and close contact with the bladder wall. Heterogeneous enhancement, and wash-out time of 54 s on CEUS (**Figure 1C**).

**Figure 2:**
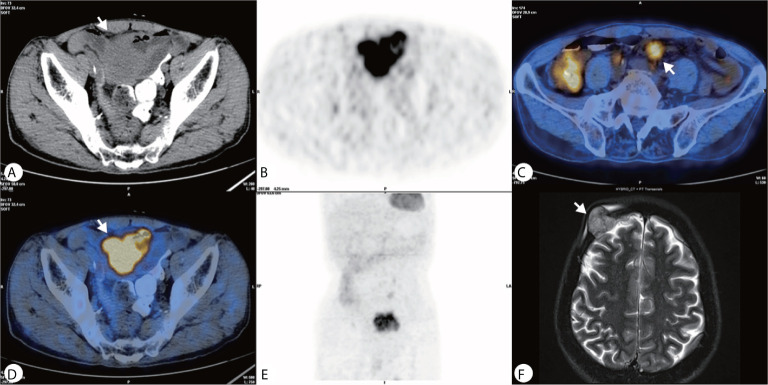
**(A–E)** PET–CT showed a heterogeneously hypermetabolic pelvic mass, and multiple hypermetabolic nodules were observed in the mesentery; **(F)** An MRI 2 months after surgery showed an irregular lesion on the right side of the frontal bone, which grew across the skull and invaded the brain tissue.

The PET–CT was performed revealing a highly metabolic mass in the lower abdomen and multiple metabolic nodules in the mesentery (**Figures 2A–E**). This was considered a small intestinal stromal tumor with lymph node metastasis. The patient did not show symptoms of lymphoma, and no abnormalities were found in other organs. The consensus of the attending radiologists and surgeons was that it was a malignant tumor rather than an inflammatory process. A core needle biopsy was rejected because of the broad area of necrotic tissue; there was also concern that adequate tumor tissue would not be obtained while risking intestinal perforation or tumor dissemination. Finally the patient underwent surgery to remove the lesion. A 5× 6 cm brown cauliflower-like mass was resected from the ileum, 50 cm away from the ileocecal junction and the surrounding lymph nodes. This mass invaded the serosal layer at the inferior portion of the bladder.

**Figure 3:**
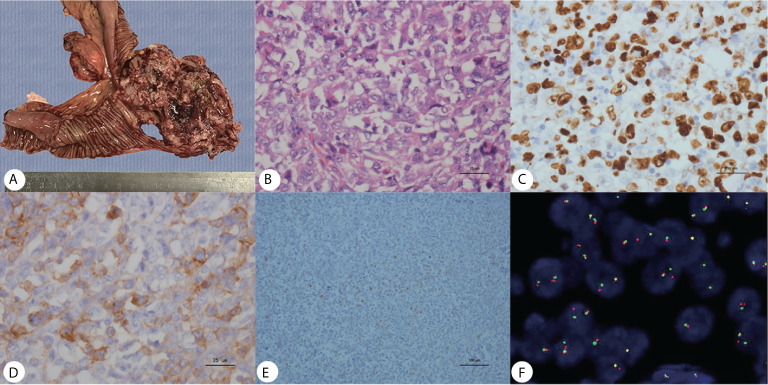
**(A)** Gross pathology revealed an ileum tumor specimen with brittle texture and multiple ulcerations on the surface; **(B)** H&E staining revealed small round blue cells; ×40. **(C–E)** Immunohistochemistry showed positive Ki67, CD99 and Fli-1 staining; ×40. **(F)** Molecular analysis revealed positive EWSR1 fusion genes.

Gross pathology showed the tumor to be friable and having multiple ulcerations on the surface (**Figure 3A**). H&E sections revealed a small, blue, round tumor (**Figure 3B**). Histopathological examination showed positive CD99, CK (pan), Ki67 (70%+), Fil-1, and CD34 levels (**Figures 3C–E**). Molecular analysis revealed positive EWSR1 fusion gene transcripts, as shown by RT-PCR (**Figure 3F**). Based on morphology and immunohistochemistry, the tumor was diagnosed as EES/PNET. The patient refused to receive chemotherapy after surgery. He was requested to come to the hospital for examination every month for the first half year, but he did not follow the advice. He came to the hospital because a soybean-like mass was on his forehead, and denied any other symptoms. An MRI was performed revealing a 2.3 × 2.1 × 2.3 cm lesion on the right side of the frontal bone (**Figure 2F**). The mass extended to the skull and invaded the brain tissue. A second surgery was performed to remove the lesion and adjacent erosive bone. Post-operative pathologic diagnosis revealed the same histology, *i.e.* the mass metastasized from the primary small intestinal tumor. We performed a telephone follow-up with this patient every two months but learned that he did not undergo any further treatment after the second surgery and died 6 months later; there was no more information about his death.

## Discussion

EES is rare, occurs mainly in adolescents, and confers a poor prognosis (11, 12). The most common primary sites of the disease are in the lower extremities (41%), pelvis (26%), chest wall (16%), upper extremities (9%), spine (6%), hands and feet (3%) and skull (2%) (12). ESS present in rare sites has been reported to occur in the atrium, esophagus, maxillary sinus, and iris (13–18). In this case, the patient is a 69-year-old male presenting with an abdominal mass, making this case double rare (19, 20). Compared to young ES patients, elderly ES patients often have a poorer prognosis (20).

The imaging features of EES are non-specific. CT mostly presents a large, well-limited mass which is relatively hypodense or isodense compared to the adjacent muscle. It could contain lower attenuation areas due to intratumoral necrosis, presenting intense and heterogeneous enhancement. On MRI, this tumor is often of low to intermediate signal intensity on T1-weighted images; of high intensity on T2-weighed images and exhibits heterogeneous contrast enhancement. Occasionally, it shows a homogeneous, moderate enhancement on contrast-enhanced T1-weighted images. On Pet/CT, it often reveals increased metabolic activity, presents heterogeneity depending on degree of internal necrosis and hemorrhage (3, 9–11, 21–23). Our case provides the US appearance of ES in the intestine; it shows a well-defined heterogeneous solid mass on ultrasound; CEUS presents a heterogeneous high-enhancement mass with irregular necrosis and fast wash-in and wash-out; it is different from the inflammatory mass which has no wash-out or slow wash-out; enhancement and wash-out patterns on CEUS indicate a malignant lesion. Therefore, The EES/PNET imaging diagnosis requires a multimodality approach and should be consciously listed as possible differential diagnoses after excluding common tumors (9, 10, 24, 25).

EES/PNET of the intestine can be easily misdiagnosed due to the fact that its clinical and imaging features are similar to other types of malignant tumors, as experienced by our patient. It should be differentiated from the most common small intestine tumors including small bowel adenocarcinomas (SBA), malignant gastrointestinal stromal tumors (GISTs), and intestinal lymphoma. 1. SBA: It has been reported the most frequent histologically malignant tumor of the small intestine; most SBA arises in the duodenum; it can also arise in the jejunum, ileum, or in unspecified location. It often occurs at 60–70 years old. The most frequent symptoms are abdominal pain, obstruction, and occult gastrointestinal bleeding. Typically SBA gives an annular constriction to the intestine and grows into the cavity; infiltration into surrounding structures and distant metastases appears early (26, 27). 2. GIST: It is a mesenchymal neoplasm that arises in the gastrointestinal tract, common in the stomach or the small intestine. It can occur at any age, but mostly reported in individuals at the median age of 60–65 years. It typically causes bleeding, anemia, pain, and seldom obstruction. It mainly presents as eccentric growth outside the intestinal cavity; large GISTs are typically soft and fragile and prone to necrosis and hemorrhage; intratumor infection can occur when the ulcer is large, but local lymph-node metastases are rare (28, 29). 3. Intestinal lymphoma: It originates in the lymphoid tissue of the bowel wall, generally occurs in the ileum, and usually has a history of extra-intestinal lymphoma. It often occurs at a younger age (10 years or over 50 years). It presents diverse symptoms, mostly anemia, pain, diarrhea, and weight loss. It is characterized by diffuse infiltration and is not confined to a small area of the intestine; the mesenteric lymph nodes appear early, but it seldom invades the surrounding organs (30, 31). In our case, according to the clinical symptoms and imaging features, the preliminarily indication was that the tumor was malignant, but it was difficult to make a clear differential diagnosis from other common malignant tumors in the small intestine. In addition to the above differentiation, EES/PNET should be differentiated from inflammatory bowel disease (IBD). In this case, CT considered inflammation with abscess formation. However, the patient has no history of IBD, and bowel wall had no imaging changes such as inflammatory edema, anti-inflammatory treatment was ineffective, therefore, the diagnosis of IBD was excluded.

EES/PNET is not only a big challenge for imaging diagnosis but also poses challenges for pathology. EES masses often present with extensive hemorrhaging and necrosis; a fine needle biopsy is usually inadequate for diagnosis (21, 32). EES/PNET is termed as the Ewing’s family since they all show characteristics of small round blue cell tumors, immunohistochemical analysis of CD99, and FLI-1 helps in diagnosing ES/PNET. Still, these markers also can be expressed in other malignant tumors such as lymphoblastic lymphoma, other round cell sarcomas, solitary fibrous tumors. ES/PNETs are characterized by specific chromosomal translocations of the EWSR1 gene (1, 21, 25, 33). The diagnosis of ES is usually made postoperatively and requires histological, immunohistochemical, and molecular techniques.

EES is clinically characterized by rapid growth of the soft tissue mass, which is often manifested early in the lung, lymph nodes, and bone metastases (23). The treatment for EES consists of surgery, chemotherapy, and radiotherapy. The 5-year survival rate of EES after surgery and chemotherapy is ~70% (1, 12). In our case involving an elderly individual with ES in the small intestine, the patient did not receive chemotherapy and had distant metastasis. Unfortunately, his survival was only 6 months after the second surgery, further proving that EES is very aggressive and has a poor prognosis in the elderly. Therefore, local surgery treatment cannot predict a favorable survival of EES in the elderly.

## Conclusion

We report a rare case of senile, small bowel primary ES showing rapid skull metastasis. A variety of preoperative imaging showed malignant features but it was difficult to distinguish it from common intestinal malignancies. Surgical resection is a conventional treatment, but due to its highly aggressive biological behavior has limited effects on improving the survival rate of EES. Thus, it is necessary to explore multimodality treatment approaches to achieve a better favorable outcome for elderly EES patients.

## Data Availability Statement

The original contributions presented in the study are included in the article/supplementary material. Further inquiries can be directed to the corresponding author.

## Ethics Statement

The studies involving human participants were reviewed and approved by Shengli Clinical Medical College of Fujian Medical University, Fujian Provincial Hospital. The patients/participants provided their written informed consent to participate in this study.

## Author Contributions

JY and HW analyzed the data and image acquisition, revised the manuscript, have contributed equally to this work. SW designed the study and revised the manuscript. All authors of this manuscript have actively participated in the data acquisition, and they all commented and approved the final version of the manuscript.

## Conflict of Interest

The authors declare that the research was conducted in the absence of any commercial or financial relationships that could be construed as a potential conflict of interest.
